# Head-Toes-Knees-Shoulders task and EF in two samples of adolescents in Brazil and United States

**DOI:** 10.3389/fpsyg.2023.1149053

**Published:** 2023-09-14

**Authors:** Valter R. Fernandes, Derek R. Becker, Megan M. McClelland, Andrea C. Deslandes

**Affiliations:** ^1^Exercise Neuroscience Laboratory, Institute of Psychiatry, Federal University of Rio de Janeiro, Rio de Janeiro, Brazil; ^2^Department of Human Services, College of Education and Allied Professions, Western Carolina University, Cullowhee, NC, United States; ^3^Hallie E. Ford Center for Healthy Children & Families, Oregon State University, Corvallis, OR, United States

**Keywords:** self-regulation, executive function, cognitive development, measurement, academic achievement

## Abstract

Executive function (EF) is a foundational cognitive construct, which is linked to better cognitive and physical health throughout development. The present study examines the construct validity of an EF task, the Head-Toes-Knees-Shoulders task (HTKS) that was initially developed for young children, in a sample of adolescents. We investigate the initial validity and range in scores between 54 adolescents from Brazil (mean age 12.58) and 56 US adolescents (mean age 12.48) from different socioeconomic contexts. Results indicated that the HTKS showed sufficient variability in both samples, especially for a measure of HTKS efficiency (completion time divided by the total score). The US sample performed better on all cognitive measures. For the Brazilian sample, regression models controlling for age and sex showed a significant relationship between the digit span working memory task, the HTKS total score, and the HTKS efficiency score. The Heart and Flowers cognitive flexibility measure was also included as an independent variable only for the Brazil sample, showing a significant relationship with both HTKS scores. For the US sample, results showed that only the HTKS efficiency score was significantly related to the digit span working memory task. This study highlights the importance of cognitive efficiency measures to achieve greater validity, as they can assess a broader range of performance with different populations. The HTKS showed good ecological validity with two adolescent samples, as it differentiated between populations with high and low socioeconomic status from different cultural contexts.

## Introduction

Recent reports document the importance of children’s self-regulation and executive function for a variety of important life outcomes such as educational success and indicators of health and well-being (McClelland et al., 2013; Robson et al., 2020). These skills lay the foundation for school success and predict long-term educational outcomes starting in early childhood and through adolescence (Duckworth et al., 2010; McClelland et al., 2013). Self-regulation consists of the capacity to consciously control thoughts, feelings, and behavior and stems from executive function (EF) skills that experience rapid growth in early adolescence and which correspond to rapid brain development during this time (Zelazo and Carlson, 2012).

EF is recognized to include the cognitive processes of working memory, inhibitory control, and cognitive flexibility (McClelland et al., 2015). It has been described in different ways by the scientific literature. Researchers have described “Hot EF” as the emotional and motivational abilities that mediate cognitive control, likely engaging the prefrontal cortex orbitomedial and ventromedial, and its connections to limbic structures (Rubia, 2011). In contrast, “Cool EF” refers to more cognitive abilities, such as working memory, inhibitory control, shifting, planning, and fluid intelligence, mainly demanding dorso and frontolateral prefrontal cortex (Zelazo and Carlson, 2012). The ability to use perception and knowledge to select actions and thoughts coherent with our emotions and motivation allows us to respond accurately to what the environment demands (Friedman and Robbins, 2022). Working memory allows individuals to remember and process information, inhibitory control includes the ability to inhibit and manage thoughts and behavior, and cognitive flexibility enables individuals to focus on a task and switch attention when needed (Blair and Ku, 2022). During development, working memory and cognitive flexibility are particularly important, showing consistent correlations across middle childhood, adolescence, and early adulthood (Best and Miller, 2010). Together, these cognitive processes allow for the top-down regulation of thinking and behavior and help individuals to follow through on instructions, demonstrate self-control, and effectively manage tasks. In the present study, we focus on a measure of EF in adolescents that requires the integration of cognitive aspects of executive functioning in a real-life behavioral situation. The Head-Toes-Knees-Shoulders (HTKS) task was developed for young children and has recently been extended to measure EF in older adults (Cerino et al., 2018) and in adolescents (Dutra et al., 2022). In this study, we analyze the HTKS and its relations to other measures of EF in a sample of adolescents in Brazil and the United States, including the variability, range in scores, and construct validity.

### The importance of EF for children and adolescents

During early childhood, EF becomes increasingly important and are crucial for children in the social environment of school and for academic success. They are described as a set of cognitive skills that are essential for social interaction, daily activities, work and learning (Diamond, 2013). A child’s ability to control and regulate their behavior predicts economic and health outcomes, and academic achievement and lowers the chance of having a criminal record (Moffitt et al., 2011).

Although most research on EF is focused on early childhood, it is a set of cognitive skills linked to several indicators of positive development between early childhood and adolescents (Best et al., 2011; Ahmed et al., 2019). Indeed, strong adolescent EF is associated with a lower body mass index (Reinert et al., 2013; Ronan et al., 2020), lower levels of media (Baumgartner et al., 2014) and substance use (Nigg et al., 2006), and better academic outcomes (Latzman et al., 2010; Samuels et al., 2016). The literature has pointed to different developmental trajectories for components of EF, but working memory may have a slower maturation process (Igazság et al., 2019). Although there is mixed evidence about whether adult-level performance is reached between 14 (Luna et al., 2004) and 17 years of age (Igazság et al., 2019), there is agreement on the rapid development of working memory and cognitive flexibility during adolescence (Luna et al., 2004; Best and Miller, 2010; Igazság et al., 2019).

Working memory and cognitive flexibility are highly involved in general and mathematical problem solving (Raghubar et al., 2010; Carden and Cline, 2015). They are critical for response inhibition and decision making (Best et al., 2011; McClelland et al., 2014; Ludyga et al., 2022), which are also required in sports tasks and real-life situations such as children’s play. The HTKS requires working memory to inhibit responses and also asks participants to switch rules using cognitive flexibility. Given the importance of working memory and cognitive flexibility in middle childhood and adolescence, the present study compared performance on the HTKS with measures of working memory and cognitive flexibility.

### Exposure to different social and educational environments and links to EF in children from demographically-diverse communities

Academic performance is a multifactorial outcome that can be influenced by several variables, such as school and family structure (O’Malley et al., 2015), school and community resources for leisure time physical activity (Centeio et al., 2018), parental education, criminality and violence levels (Ruediger, 2017). Unfortunately, children who do not get their basic needs met in their social and family environments are more likely to face challenges in their cognitive development (Lipina and Segretin, 2019). In Brazil, as in other countries, educational and income inequalities are strongly correlated (de Souza and De Carvalhaes, 2014). Low levels of parent education can reduce a child’s opportunity to receive a quality education and achieve a higher education and income level (Medeiros et al., 2020). For example, in Brazil, children that live in low SES neighborhoods in Rio de Janeiro often suffer from high levels of criminality, which can affect their mental health and negatively impact academic performance (de Moraes et al., 2021). Nonetheless, even in neighborhoods with higher levels of criminality and violence, children’s EF can be positively impacted by school interventions (Watts et al., 2018).

In the United States (US), educational and income inequalities are also strongly correlated and predict lower EF and academic outcomes (Hackman and Farah, 2009; Ahmed et al., 2019). Neighborhood risk factors, such as high rates of crime and limited access to positive community resources (e.g., parks and green spaces) are linked to low levels of school success and poor health (Dadvand et al., 2015; Yu and Lippert, 2016; Wende et al., 2021). Children growing up in rural settings in the US, such as those in the present paper, are also at increased risk for low levels of physical activity and high rates of obesity (Joens-Matre et al., 2008; Gunter et al., 2015). Brazil’s Human Development Index (HDI), adjusted for inequality, is medium, while the US is classified as very high. This characterizes a large difference between the two countries in terms of the social context for children’s development [UNDP (United Nations Development Programme), 2022]. These findings highlight the importance of using sensitive and ecologically valid measures to assess children’s EF in different contexts.

### Measures of EF in childhood and adolescence

Several EF assessments have been developed and adapted to capture better ecological validity and for children to be assessed in naturalistic rather than laboratory settings (Watts et al., 2018; Diamond et al., 2019; Gentile et al., 2020). Computerized EF tasks usually alternate between congruent and incongruent visual stimuli, with rules that alternate according to such stimuli. For example, the Hearts and Flowers test uses this principle, requiring working memory to maintain the rules related to each visual stimulus, inhibiting responses and cognitive flexibility for more accurate decisions in incongruent tasks (Wright and Diamond, 2014). Such tasks permit the registry of different measures of accuracy and reaction times in the different conditions, as well as in blocks that alternate between incongruent and congruent tasks.

Another popular EF measure, the Stroop test, requires saying colors in different conditions. As in the Heart and Flowers measure, an interference cost variable has been calculated and used by several authors (Lee et al., 2019). Another way to analyze performance in EF tests is cognitive efficiency, defined as the increase in knowledge gained concerning the amount of time invested in its acquisition (Hoffman, 2012). In this way, researchers have looked for ways to interpret scores, including a view that includes both accuracy and reaction time scores. For example, cognitive efficiency measures have been calculated considering the number of accurate responses per minute (Coffman et al., 2017; Miller et al., 2019). Lower scores indicate better performance as they represent lower costs of time to produce correct answers. In the present study, to better capture variability in the HTKS with adolescents, a measure of HTKS efficiency was created using the HTKS completion time (Cerino et al., 2018) and dividing it by the total HTKS score. We used both the HTKS efficiency score and the HTKS total score to provide a broader understanding of the variability and cognitive performance measured by the HTKS.

### Different measures for the HTKS task for different populations

Given the association between EF with long-term health and cognitive development it is important to understand its developmental trajectory from early childhood into adolescents. The work that has assessed longitudinal measurement invariance with tasks that assess EF (Willoughby et al., 2012; Ahmed et al., 2019), has often employed assessments that require special software and a time-consuming application. This restricts how and where the assessment can be given.

The Head-Toes-Knees-Shoulders (HTKS) task is a brief and easy-to-administer measure of EF that has been extensively used with preschool and kindergarten-age children (Cameron Ponitz et al., 2007; McClelland et al., 2014; Kenny et al., 2023). The HTKS demonstrates strong reliability and validity (McClelland et al., 2014) and is related to academic and health-related outcomes during early childhood (Cameron Ponitz et al., 2009; Becker and Nader, 2021). Although the HTKS was primarily developed for younger children, recent work has extended the use of the task to older populations.

In a study with children in middle childhood and early adolescence, Dutra et al. (2022) analyzed the construct validity of the HTKS with measures of short-term memory and working memory, finding both tasks related to higher HTKS total scores. However, results also showed potential ceiling effects and low variability that might have impaired the construct validity of the HTKS. A related study with older adults used completion time on the HTKS and found evidence of construct validity with significant correlations with the NIH Toolbox and stronger correlations using the completion time variable relative to the HTKS total score (Cerino et al., 2018). Using a measure of cognitive efficiency could help avoid potential ceiling with the HTKS and be more closely aligned with traditional EF assessments used with older children.

The HTKS challenges executive control through incongruent motor responses to stimuli, and asks children to do the opposite to a given command. The assessment requires all aspects of EF – working memory to remember the rules, inhibitory control when asked to do the opposite of a command and cognitive flexibility when the rules change. The present study will build on the work of Dutra et al. (2022) and Cerino et al. (2018) by developing a measure of efficiency to help improve the sensitivity of the HTKS for use with older populations.

In the present study, we examined range in scores and construct validity of the HTKS in two samples of adolescents in the U.S. and Brazil. We extended the scoring on the HTKS, and included a measure of HTKS efficiency, which was the HTKS completion time (Cerino et al., 2018) divided by the total HTKS score. In addition, we analyzed the differences in the HTKS measures between both samples. In developing countries, there are more high school students who repeat grades, which can lead to greater age variability during the school years. This highlights the potential importance of using the HTKS efficiency measure (which may be more comprehensive and have greater variability) given the expected heterogeneity between the two samples in Brazil and the US.

We hypothesized that both samples of adolescents would show variability in the HTKS and EF measures. The HTKS efficiency score was expected to demonstrate stronger correlations with a measure of working memory than the HTKS total score. Differences between Brazilian and US children’s performances were expected since both countries are in different socioeconomic and cultural environments, and this could impact children’s cognitive development and performance. Considering these different contexts, we expected lower scores in the Brazilian sample.

## Methods

### Participants

This study examined the variability in scores and initial validity of the HTKS in adolescents in the United States (US) and Brazil. Participants included a combined 111 children who ranged in age between 10- to 15-years of age. We performed a post-hoc G-power sample size analysis, which showed that both samples could achieve significant results for an arbitrary target power of 95%.

### Brazil sample

The Brazilian sample consisted of 55 students (65% male) from public schools aged 10 to 15 years, taken from a longitudinal study focused on the effect of physical exercise interventions on children’s cognition. Fifty-four percent of them were in grade six (mean age 12.58), 43% were in grade seven (mean age 12.91), and 4% were in grade eight (mean age 13.50). Participants and their guardians were informed about the study and gave informed consent. Children were excluded from this study if they had any hearing or visual impairments. Physical evaluations and HTKS were performed and computerized cognitive assessments were carried out on a subsequent day. This project was approved by the Ethics and Research Committee of the *Centro Universitário Augusto Motta* (UNISUAM) (CAAE 11980119.4.0000.5235).

### United States sample

The U.S. sample included 56 adolescents (60% male) between the ages of 11 and 14 years attending a public lab-based middle school (grade 6 through grade 8) in the Southeast region of the U.S. The lab-based middle school was run by the public Regional University in collaboration with the local school district. Parents gave permission for their children to participate in research as a part of attending the lab school with the understanding that their child could opt out of any study. Fourteen percent of participating adolescents were in grade six (mean age 11.2), 42.5% were in grade seven (mean age 12.5), and 41.5% were in grade eight (mean age 13.5). This project was approved by Western Carolina University IRB PROJECT TITLE: [1156016–4] Catamount H.I.I.T.

#### Overall EF measure

##### Head toes knees shoulders (HTKS)

Children from the U.S. and Brazil both participated in the HTKS. The Portuguese version was used in the Brazilian sample and the English version was used in the US. The HTKS has 17 practice items and 30 test items that measure EF (working memory inhibitory control, and cognitive flexibility) with high ecological validity (McClelland et al., 2014, 2021). The task includes three sections. In part one, children have to touch their heads when instructed to touch their toes and, touch their toes when instructed to touch their heads. Part two includes a new command pairing shoulder with knees. In part 3, the four paired commands are reversed (head = knees, shoulders = toes). Correct responses are scored as a 2, self-correct are scored as a 1, and incorrect responses receive a 0.

The HTKS efficiency variable was calculated by combining accuracy and the time to complete the HTKS [(time/accuracy)/100]. The efficiency variable was created by first obtaining a total score on the HTKS by summing the three parts (range 0–60). The time variable assessed the amount of time in seconds children took to complete the HTKS using a digital stopwatch. The stopwatch was started at the point where the HTKS administrator began reading the instructions and stopped once the assessment was complete. Lower HTKS efficiency scores indicate better EF.

#### Working memory

##### Digit span task (forward-order and reverse-order)

In Brazil and the US, adolescents were administered either a computerized version (Brazil) or a paper and pencil version (US) of the digit span working memory task. The Forward Digit Task tests short-term memory and serves as an introduction to the Backward Digit Task, which is considered a working memory task. The sum of the two sub-items has been described as a measure of working memory (Gentile et al., 2020), with a high degree of reliability and validity (Conway et al., 2005). In both steps, participants hear the digit strings and need to retrieve them (directly or inversely) by selecting the digits from a circle of digits by touching the touchscreen. Depending on performance, participants move up or down a level. The evaluation ends after 14 attempts. The measure of the maximum length of up to two errors of the reverse order phase responses was used as a working memory measure (Woods et al., 2011). For adolescents in the US, the task was given in the same format as the computerized task completed by the Brazilian children, with the forward-order digit task followed by the reverse-order task. Adolescents in the US sample gave verbal responses. The task took approximately 5 minutes to complete and was administered in a quiet location.

#### Cognitive flexibility

##### Hearts and flowers task (H&F)

In Brazil, participants completed the Hearts and Flowers Task (H&F) and received information from congruent and incongruent tasks. When a heart appears on the screen, it must touch the same side as it and when a flower appears, it should touch on the opposite side. The H&F task includes the following phases: blocks with only hearts, blocks with only flowers, and blocks with a mix of hearts and flowers. All blocks had an initial moment of task learning, in which feedback was provided for each child’s response, reminding them, in case of error, of the task objective (congruent in the case of the apparition of the heart, and incongruent in the case of the flower apparition). In this study, we use the mixed block phase as a measure of cognitive flexibility (Stein et al., 2017), reaction time and accuracy were registered (Davidson et al., 2006) and used to calculate the H&F efficiency score [(time/accuracy)/100]. The H&F task is validated and widely used in the evaluation of children’s executive function in different countries (Spiegler and Leyendecker, 2017; Watts et al., 2018; Rosas et al., 2019).

##### Analytic plan

Descriptive statistics, tests of normality, and validity analyzes were completed separately in Stata 17 for the US and Brazilian samples. Normality was assessed by estimating skewness and kurtosis for both the HTKS and HTKS efficiency scores. Differences in HTKS measures between both samples were analyzed using independent samples t-tests. Bivariate correlations were used to assess validity for all variables of interest and multivariate regression, controlling for age and sex, were assessed separately for the HTKS and HTKS efficiency scores.

The percentage of missing data was small and was specific to age for the U.S. sample (*n* = 9, 17%) and Hearts and Flowers for the Brazilian sample (*n* = 3, 6%). Full Information Maximum Likelihood estimation (FIML) (Schafer and Graham, 2002) was used to appropriately address missing data for all regression models with the US and Brazilian samples.

## Results

All research questions including descriptive statistics were analyzed for both samples. Table 1 includes the means and standard deviations for all the study variables, as well as the comparison of the results between the groups of Brazilian and US children. Measures of skewness and kurtosis are displayed in Table 2 with correlations displayed in Table 3.

**Table 1:** Descriptive analysis of the Brazilian and US children at baseline.

	Brazil N 54 (19 female, 35 male)		US N 56 (22 female, 34 male)		*t*	*p*	*Cohen’ d*
	M (SD)	Min – Max	M (SD)	Min – Max			
Age (years)	12.58 (0.89)	11.10–15.3	12.48 (0.85)	11–14	0.53	0.59	0.10
HTKS	46.77 (6.44)	32–57	55.62 (5.43)	38–60	7.79	<0.001	1.49
HTKS Efficiency	62.08 (12.03)	26.38–57.71	51.39 (10.79)	33.40–82.90	−4.90	<0.001	0.94

HTKS (Head, Toes, Knees and Shoulders); HTKS Efficiency (Mean reaction time divided by accuracy, divided by 100).

**Table 2:** HTKS ceiling effect analysis.

		*N*	Mean (SD)	*p*-value Skewness	*p*-value Kurtosis	Joint test *p* > *χ*2	Ceiling%
Brazil	HTKS	54	46.78 (6.45)	0.24	0.08	0.10	1.99%
	HTKS efficiency	54	62.08 (12.03)	0.004	0.39	0.02	1.99%
US	HTKS	56	55.62 (5.43)	0.00	0.16	0.00	39.29%
	HTKS efficiency	56	51.39 (10.79)	0.01	0.21	0.03	3.58%

BR, Brazil; US, United States.

**Table 3:** Pearson correlations.

		Age	Digit FB
HTKS	BR	−0.26^†^	0.33*
	US	0.19	0.10
HTKS efficiency	BR	0.17	−0.28*
	US	−0.26^†^	−0.25^†^

Digit FB (digit forward plus backward); BR, Brazil; US, United States. Significance levels: ^†^ < 0.1, *p < 0.05.

### Does the HTKS demonstrate sufficient variability and range in scores in two samples of adolescents in Brazil and the US?

**Figure 1:**
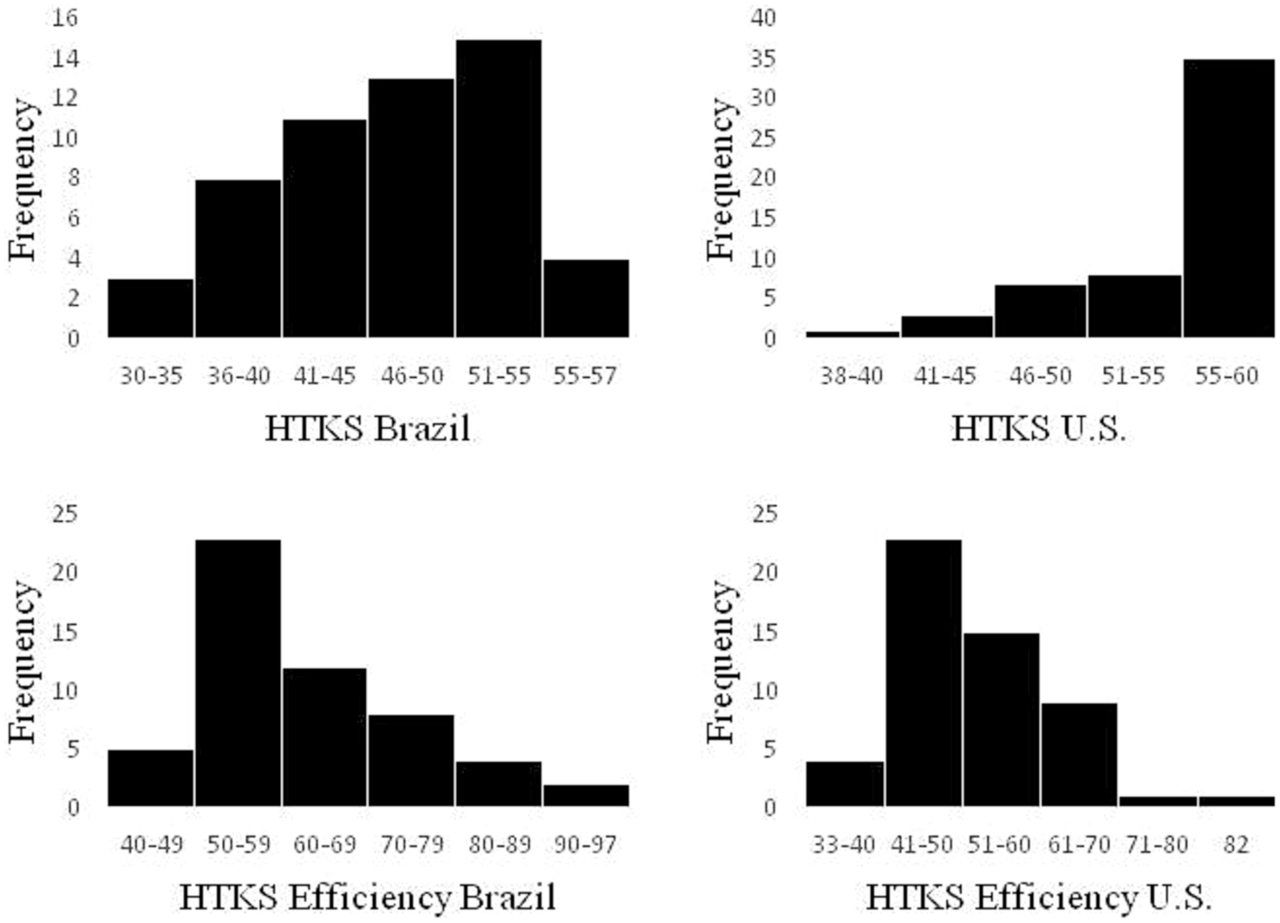
Histograms for HTKS total score on top and HTKS Efficiency on the bottom, for United States and Brazilian samples.

Results showed that HTKS scores demonstrated sufficient variability and range in scores for both samples, especially when using the HTKS efficiency score. For the total HTKS score and the HTKS efficiency score, 2% of Brazilian children scored at the ceiling. Brazilian children showed normally distributed HTKS scores and a significant positive skew for the HTKS efficiency score (see Table 2; Figure 1). In the sample of US children, 39% scored at the ceiling with the total HTKS score and less than 4% of US children scored at the ceiling with the efficiency score. Scores on the HTKS were significantly negatively skewed and scores on the HTKS efficiency measure were significantly positively skewed for US children (Table 2 and Figure 1).

### Does exposure to different social and educational environments influence EF performance?

T-tests showed significant mean differences on the HTKS, with the U.S. sample showing better performance on both the HTKS and HTKS efficiency scores. However, no significant mean differences were found for age and the distribution of boys and girls between Brazilian and US samples.

### Does the HTKS demonstrate initial construct validity in two samples of adolescents in Brazil and in the US?

We looked at the initial validity of the HTKS in samples of adolescents in the US and Brazil. To test HTKS construct validity, we first analyzed correlations between the measures of working memory collected in both samples (digit span) with both the HTKS and HTKS efficiency scores in both samples (see Table 3).

Results indicated significant correlations between measures of working memory and EF expressed by accuracy (HTKS total score) and a measure of cognitive efficiency (HTKS efficiency), but only for the Brazilian sample. As can be seen in Table 3, for Brazilian children, the HTKS and digit span task showed a small positive correlation (*r* = 0.33, *p* < 0.05), with the HTKS efficiency task significantly inversely correlated with the digit span task (*r* = −0.28, *p* < 0.05). The correlations between the digit span and the HTKS and HTKS efficiency score were non-significant for US children, but the correlation between the digit span and the HTKS efficiency score approached significance (*r* = −0.25, *p* < 0.10) and was similar in magnitude to the correlation in the Brazilian sample.

The Heart and Flower efficiency score was also included as an additional dependent variable for the Brazilian sample as a measure of cognitive flexibility. Results showed significant positive correlations for the HTKS total score (*r* = −0.36, *p* < 0.01) and the HTKS efficiency score (*r* = 0.30, *p* < 0.05) with the Heart and Flower efficiency score.

To further assess the HTKS construct validity, a series of regression models were run with digit span as the dependent variable for both Brazilian and US children. All models controlled for age and sex. For the Brazilian sample, a significant positive relationship (HTKS, ß = 0.36, *p* = 0.024) and a significant negative relationship (HTKS Efficiency, ß = −0.34, *p* = 0.019) were found for the digit span task. Results for the Heart and Flower efficiency score in the Brazil sample were also significant and in the same direction for the HTKS (ß = 0.29, *p* = 0.047) and the HTKS efficiency score (ß = −0.41, *p* = 0.012). Results for the HTKS and HTKS efficiency score for the U.S. sample were in the same direction as the Brazilian sample, but only the HTKS efficiency score was significantly negatively related to the digit span task (HTKS efficiency, ß = −0.29, *p* = 0.024, see Table 4).

**Table 4:** Linear regressions controlling for sex and age.

	Predictors	B	SE	*ß*	*R* ^2^	*R*^2^ adjusted	*p*
Digit FB Brazil	HTKS efficiency	−0.039	0.016	−0.34	0.108	0.055	0.019*
	HTKS score	0.07	0.03	0.36	0.10	0.047	0.024*
Digit FB US	HTKS efficiency	−0.08	0.03	−0.29	0.08	0.02	0.03*
	HTKS score	0.06	0.07	0.11	0.01	0.00	0.38

Digit FB (digit forward plus backward). Significance levels: *p < 0.05.

## Discussion

In this study, we investigated the initial variability and range in scores for Brazilian and US adolescent children on the HTKS, a well-validated measure of EF used in preschool and kindergarten populations. We also expanded past work with the HTKS (e.g., McClelland et al., 2014; Kenny et al., 2023) by creating an efficiency score, reducing the potential ceiling effects found with older populations. Results indicated that there was sufficient variability and range in HTKS efficiency scores, with the Brazilian and US sample scoring at the ceiling for only 2 and 4%, respectively. For the HTKS total score, 39% of the US children scored at the ceiling, while only 4% of Brazilian children scored at ceiling levels. It has been suggested that psychometric instruments must avoid ceiling and floor effects in order to produce more reliable measures (Diamond, 2012). The Dutra et al. (2022) study included a Brazilian sample of adolescents, and the mean HTKS total score approached the ceiling. This was cited as a possible reason for the small correlations found in the study. Ceiling or floor effects may prevent the detection of significant patterns of correlations among different cognitive measures, especially in children from developing countries (Spiegel and Lonigan, 2018; Gonzales et al., 2021). In this sense, the HTKS efficiency score, which indicates faster processing for accurate answers and better cognitive functioning, proved to be a more satisfactory measure for both samples in the present study.

The T-test results reported significant differences between both samples and significantly lower performance in the Brazilian sample. This finding corroborates previous literature that has reported lower levels of EF in children from low socioeconomic status countries (Sarsour et al., 2010; Lipina and Segretin, 2019). Crowded home environments and dangerous neighborhoods may contribute to lower EF skills (Ribeiro et al., 2016).

Second, we examined whether the HTKS was associated with measures of EF in two samples of adolescents, from Brazil and the US. We found small significant correlations between measures of EF (working memory), in the HTKS total score and when using the measure of HTKS efficiency with the Brazilian sample. This was not the case with the American sample, which showed a trend in the correlation for the HTKS efficiency measure (*p* < 0.1) although the magnitude of the correlation was similar to that of the Brazilian sample. These results could be explained by the larger variability with lower scores in Brazil compared to the US and the better educational and socioeconomic environment in the US.

Regression analyzes that controlled for sex and age indicated that the HTKS efficiency score was negatively and significantly related to the digit span measures in both Brazil and US samples. Shorter time spent on accurate responses was significantly related to better working memory. The importance of analyzing cognitive efficiency was corroborated by the results with the Heart and Flower score as the dependent variable in the Brazil sample. The measures of accuracy and efficiency of the HTKS were correlated with Heart and Flower efficiency, but the stronger correlation was with the HTKS efficiency score. The HTKS efficiency score was consistently related to working memory, and with the Heart and Flower task, which is a measure of cognitive flexibility. At school, cognitive efficiency is crucial in learning in classrooms, where there can be a lot of content covered in a limited time (Hoffman, 2012). These results show that scores on the HTKS were related to scores on measures of WM and cognitive flexibility and give initial support for the construct validity of the HTKS in older children in the US and Brazil.

### Implications for research and practice

Our study highlights the importance of using measures of cognitive efficiency with older children and supported the initial validity of a measure of EF with strong ecological validity (the HTKS) to other measures of EF (e.g., working memory and cognitive flexibility). Researchers and educators can assess cognitive performance in a naturalistic setting, with an easy-to-use and psychometrically valid measure of EF. In addition, results supported using the HTKS in diverse populations from two different countries, showing that it can be a useful tool to be applied in low and high socioeconomic status countries.

Although the study had a number of strengths, there were also limitations. First, although the study was one of the first to look at the initial reliability and validity of the HTKS with adolescents, the sample sizes for the Brazil and US samples were small, and this limited the power to detect effects. Future research should replicate these findings with larger samples from multiple cultural contexts. Second, the digit span task used in the Brazilian sample was computerized with the US sample using a pencil and paper version. However, in both samples, the two error maximum length digit span was used in the analysis. Third, both samples had a lack of additional demographic data, such as family socioeconomic status, and ethnicity making it difficult to draw conclusions about the social context that could be influencing the outcomes. Inferences and tests of construct validity in the US sample are limited by the lack of an additional measure of EF, such as the Heart and Flowers in the Brazilian sample.

## Conclusion

Results support the variability and initial validity of the HTKS in two samples of adolescents from Brazil and the US. Including a cognitive efficiency score in the HTKS provided greater validity compared to the total HTKS accuracy score. This allowed the HTKS to assess a broader range of performance in diverse populations of adolescents. Lower educational and socio-economic contexts can negatively influence children’s EF, however the HTKS efficiency score provided sufficient range to assess performance for both populations. Future studies should investigate connections among the HTKS efficiency score with different EF assessments and with measures of academic performance.

## Data availability statement

The original contributions presented in the study are included in the article/supplementary material, further inquiries can be directed to the corresponding author.

## Ethics statement

The studies involving humans were approved by Comitê de Ética e Pesquisa do Centro Universitário Augusto Motta (UNISUAM) (CAAE 11980119.4.0000.5235). The studies were conducted in accordance with the local legislation and institutional requirements. Written informed consent for participation in this study was provided by the participants' legal guardians/next of kin.

## Author contributions

VF lead efforts on conceptualizing, writing, reviewing results, and revising drafts. DB lead efforts on data analyzing and writing the results section. MM contributed to conceptualizing, writing, and reviewing drafts. AD assisted with data analyzes, methods, and reviewing drafts. All authors contributed to the article and approved the submitted version.

## Funding

The study was financed in part by the Coordenação de Aperfeiçoamento de Pessoal de Nível Superior - Brasil (CAPES) - Finance Code 001. Participants and their parents were informed about the experimental procedure and signed an Informed Consent Form (ICF) and Statement of Consent.

## Conflict of interest

The authors declare that the research was conducted in the absence of any commercial or financial relationships that could be construed as a potential conflict of interest.

## Publisher’s note

All claims expressed in this article are solely those of the authors and do not necessarily represent those of their affiliated organizations, or those of the publisher, the editors and the reviewers. Any product that may be evaluated in this article, or claim that may be made by its manufacturer, is not guaranteed or endorsed by the publisher.
